# Supplementary material for: FAST and Agile–the MASLD drift: Validation of Agile 3+, Agile 4 and FAST scores in 246 biopsy-proven NAFLD patients meeting MASLD criteria of prevalent caucasian origin
Source: PLoS One. 2024 May 23;19(5):e0303971. doi: 10.1371/journal.pone.0303971 (PMC11115280; doi:10.1371/journal.pone.0303971)
Supplement: Attachment — Submitted filename: Platon_Monica_Protocolul studiului RO.pdf [file pone.0303971.s002.pdf]

Evaluarea prognosticului pacienților cu NASH prin elastografie ultrasonografică asociată cu analiza veziculelor extracelulare (ULYSES)

Introducere

„Boala de ficat gras non-alcoolică” (NAFLD) este manifestarea hepatică a sindromului metabolic. Estimările bazate pe datele actuale indică o creștere cu 21% și, respectiv, 63% a incidenței NAFLD și NASH până în 2030. În plus, incidența cirozei cauzate de NAFLD, carcinom hepatocelular (HCC) și deces va crește cu 168%, 137% și respectiv 178% până în 2030. În prezent, NAFLD/NASH presupune un diagnostic histopatologic, iar biopsia hepatică (LB) rămâne pentru moment standardul de aur pentru diagnostic și stratificarea riscului.

Designul studiului

- longitudinal prospectiv (cohortă prospectivă) – 93 subiecți

- cohortă retrospectivă – 300 subiecți (din 01.01.2007)

**FINANȚATOR**	Unitatea Executivă pentru Finanțarea Învățământului Superior, a Cercetării, Dezvoltării și Inovării – Proiect PN-III-P4-PCE-2021-1474
**LOCAȚIA STUDIULUI**	Universitatea de Medicină și Farmacie “Iuliu Hațieganu” Cluj-Napoca – Clinica Medicală III, Centrul de Cercetare în Medicină Avansată și Medicină Translațională MedFUTURE
**DURATA**	01.01.2007 – 31.12.2024 pentru inrolarea pacientilor (retrospectiv + prospectiv)<br>01.08.2022 – 31.12.2024 – finantarea pentru studiul prospectiv prin proiectul PN-III-P4-PCE-2021-1474
**DIRECTOR STUDIU**	Prof. Dr. Monica Platon (Lupșor)

**OBIECTIV PRINCIPAL**	Identificarea unui algoritm „multiomic” bazat pe semnături proteomice și metabolomice, imagistică inovatoare pentru diagnosticul și prognosticul pacientilor cu boala de ficat gras non-alcoolica (creștere cu cel puțin 30% față de standardul de diagnostic actual neinvaziv).
**OBIECTIVE SECUNDARE**	(O1). Testarea si validarea unei metode de diagnostic, prognostic și monitorizare pentru pacienții cu NAFLD, concentrându-se pe „5P”: (1) diagnostic potențial al NASH avansat, (2) predicția evoluției de la NAFLD la NASH, (3) predicția debutului HCC, (4) evaluarea prognosticului la pacienții cu NASH avansat, (5) predicția rezultatelor pe termen scurt (comorbidități, complicații asociate NAFLD). (a) Evaluarea și validarea performanței tehnicii 2D-SWE - disponibilă pe Supersonic Mach 30 pentru evaluarea fibrozei, steatozei si inflamatiei în NAFLD, în comparație cu VCTE + CAP și biopsia hepatica. (b) Stabilirea criteriilor tehnice pentru o mai bună evaluare a steatozei, fibrozei și inflamației prin 2D-SWE. (c) Validarea sondei XL FibroScan® în diagnosticarea NAFLD în populația din România, (d) Validarea scorurilor Agile 3+, Agile 4 si FAST score la polulatia din Romania.<br><br>(O2) Dezvoltarea de semnături proteomice și metabolomice bazate pe investigarea extraveziculelor celulare in NAFLD, seturi de date de antrenament și seturi de date de validare pentru fiecare dintre semnăturile care se concentrează pe „5P”, așa cum este descris mai sus și translatarea semnăturilor proteomice și metabolomice în practica clinică prin combinarea datelor de tip „omics” cu metode imagistice inovatoare pentru un diagnostic NAFLD de precizie.<br><br>(O3) Identificarea, izolarea și caracterizarea cu succes a veziculelor extracelulare totale și specifice hepatocitelor pentru a descoperi noi biomarkeri – analiza de identificare (categorii 1, 2) și de puritate (categoria 3) în conformitate cu Ghidurile MISEV2018 (Informații minime pentru studiile veziculelor extracelulare, 2018).

	(O4) Asigurarea sustenabilitatii proiectului și urmărirea pe termen lung pentru cohorta prospectiva prin înscrierea tuturor subiecților la examinări periodice și servicii de biobanking (înscrierea în registrul regional NAFLD).
**ACTIVITĂȚI**	**ETAPA 1. Inițierea proiectului**<br>Activitatea A1.1. Stabilirea cadrului (stiintific, etic si organizational), realizarea organigramei, a fiselor de post, a protocolului de studiu, obtinerea aprobarilor din partea comisiilor de etica, desfasurarea achizitiilor esentiale, stabilirea reguli dlore codare pentru baza de date si registru.<br>Activitatea A4 Management si diseminare<br><br>**ETAPA 2. Studiu prospectiv**<br>Activitea A2.1 Realizarea inrolarii, a evaluarii baseline si codificarea datelor si managementul bazei de date<br>Activitatea A2.2 Efectuarea TE + CAP, 2D-SWE pentru stabilirea diagnosticului si prognosticului NAFLD<br>Activitatea A2.3 Izolarea, purificarea si caracterizarea exozomilor<br>Activitatea A2.4. Efectuarea analizelor metabolomice si proteomice pentru EVs NAFLD<br>Activitatea A2.5 Realizarea analizei statistice pentru datele metabolomice si proteomice. Identificarea biomarkerilor tinta<br>Activitatea A4 Management si diseminare<br><br>**ETAPA 3. Studiu retrospectiv**<br>Activitatea A3.1 Biobanking, urmarire, codificare date<br>Activitatea A3.2 Validarea rezultatelor prin ELISA si Western Blot<br>Activitatea A3.3 Validarea scorurilor bazate pe elastografie ultrasonografica (Agile 3+, Agile 4 si FAST Score) la populatia din Romania

	Activitatea A3.4 Realizarea analizei statistice<br>Activitatea A4 Management si diseminare
**PACIENȚI**	93 pacienți înrolați în cohorta prospectivă<br>300 pacienți înrolați în cohorta retrospectivă
**CRITERII DE INCLUDERE**	1. Consimțământ informat semnat<br>2. Vârsta cuprinsă între 18-80 ani<br>3. Steatoză hepatică evaluată ecografic<br>4. Rigiditate hepatică evaluată cu aparatul Fibroscan<br>5. Steatoza hepatică evaluată prin determinarea parametrului de atenuare controlată (Fibroscan)<br>6. Efectuarea biopsiei hepatice – transjugular sau percutanat
**CRITERII DE EXCLUDERE**	1. Altă boală hepatică<br>2. Consum de alcool: bărbat >30 g/zi, femeie >20 g/zi<br>3. Malignitate, alta decât HCC<br>4. Boli inflamatorii
**DESIGNUL STUDIULUI**	**1. STUDIU PROSPECTIV**<br>Criteriile standard de diagnostic actuale (SoD) arată o acuratețe redusă pentru diagnosticul neinvaziv al NAFLD (aproximativ 40%). Presupunem că SoD combinată cu abordarea „multiomică” va putea îmbunătăți acuratețea diagnosticului până la cel puțin ~70%. Calculând că studiul va avea o putere de 0,80, un abandon de 10% şi valori p <0,05, pentru a ne testa ipoteza, va trebui să includem 93 de pacienți în brațul activ al studiului.<br><br>**Definirea grupurilor de studiu:**<br>Grupul de control (G1) – subiecți admiși la Medicală III pentru alte afecțiuni medicale, la care fibroza și steatoza sunt excluse (rigiditate hepatica <5,2 kPa), (fără steatoză, CAP <248 dB/m), sunt de acord să participe la studiu, să semneze consimțământul informat și să fie de acord cu prelevarea de sânge.

	Grupul 2 (G2) cu steatoza hepatica (CAP>248 dB/m), dar NASH cu siguranță improbabilă (rigiditate hepatica <7 kPa) care efectuează intervenții chirurgicale la Medicală III pentru alte afecțiuni medicale precum colecistectomia, chirurgia bariatrică etc., sunt de acord cu participarea la studiu, semnarea consimțământului informat și efectuarea biopsiei hepatice în timpul intervenției chirurgicale<br><br>Grupul 3 (G3) Pacienți cu NASH care efectuează biopsie hepatica, diagnosticați inițial cu steatoza hepatica (CAP>248 dB/m) + rigiditate hepatica (FS) >7 kPa. Dacă NASH nu este confirmată pe biopsie, aceștia vor fi incluși în G2.<br><br>Grupul 4 (G4). NASH cu boală hepatică compensată avansată (F3, F4).<br><br>**2. STUDIU RETROSPECTIV**<br>300 subiecți care au efectuat biopsie hepatică in cadrul clinicii Medicală III.<br><br>**Urmărire pe termen lung.** Includerea subiecților în registrul regional NAFLD: Intenționăm să includem subiecții în registrul regional al NAFLD– unul dintre proiectele colectivului Medicală III, care a beneficiat de finanțare anterioară. Intentionam sa validam scorurile Agile 3+, Agile 4 si FAST score pentru populatia din Romania
**METODE**	**1. Prelevarea de sânge -** Ser si plasma.<br><br>**2. Izolarea exozomilor.** Cromatografia de excludere prin mărime și ultracentrifugarea în gradient (centrif. (1) 300 g, 10 min, (2) 2000 g, 20 min și 1000000 g, 70 min).<br><br>**3. Caracterizarea exozomilor.** Citometrie în flux (pentru a separa EV-urile de proteinele serice circulante, contaminanți și lipoproteine, în special

	lipoproteine de înaltă densitate, etc. și oferă mostre EV mai pure), microscopie electronică (EM) microscopie cu forță atomică (AFM) după un proces de uscare a probei - acoperire cu anticorpi folosind foi de mica, analiză în modul de aer, înregistrarea imaginii în mod intermitent. modul de contact (IC-AFM) la o dimensiune de 1×1 µm2, analiză topografică prin nanoscop și software specific și împrăștiere dinamică a luminii - cantitate, tip și dimensiune.<br><br>**4. Semnătura „proteomică"** – explicată pe scurt în (Figura 1). După izolare și caracterizare, profilarea proteomului EV a peptidelor preparate cu granule paramagnetice va fi realizată prin nano-LC UDMSE așa cum sa făcut anterior (11). Extracția proteinelor se va face prin metoda freeze taw și după determinarea concentrației proteinelor, 4 ug de proteină/probă vor fi supuse analizei. Digerarea triptică după reducere și alchilare va fi folosită utilizând protocolul pe bază de perle. Pentru cuantificarea absolută a proteinelor vor fi utilizate peptide standard Hi3 E. coli (chaperonă de dezagregare a proteinelor ClpB, Waters Corporation). Un standard global de proteine va fi pregătit și măsurat pentru controlul calității după fiecare lot de 15 probe. Datele vor fi achiziționate în modul de achiziție independent.<br><br>5. **Semnătura „metabolomică"** – explicată pe scurt în (Figura 2). În primul rând, va fi implementată o abordare metabolomică non-țintită (UHPLC-MS) pentru descoperirea metaboliților semnificativi statistic între grupurile studiate. Prepararea specifică a probei (16) va fi utilizată pentru extracția metaboliților polari și nepolari. Fracțiile vor fi analizate folosind cromatografia de interacțiune hidrofitică (HILIC) – pentru fracția polară – și cromatografia în fază inversă (RP) – pentru fracția nepolară – cuplată la spectrometrie de masă de înaltă rezoluție (17).

	6. **Validarea semnăturilor proteomice prin Western Blot și ELISA**. DuoSet ELISA (R&D System) împreună cu DuoSet Ancillary Reagent Kit (R&D Systems), respectiv conjugați de anticorpi secundari specifici.<br><br>7. **TE cu CAP inclusiv sonda XL pentru diagnosticul neinvaziv și gradarea fibrozei și respectiv steatozei**. Achizițiile FibroScan® vor fi efectuate folosind sondele M și XL sub imagine ecografică asistată pentru a examina un segment de 6 mm grosime și lipsit de structuri vasculare mari din lobul drept al ficatului situat între coaste. CAP va măsura atenuarea ultrasonică în ficat folosind semnale dobândite de sonda FibroScan® pe baza elastografiei tranzitorii controlate de vibrații (VCTE) (dB/min). 2D-SWE pentru diagnosticul neinvaziv al NAFLD folosind SuperSonic Mach 30; 2D-SWE măsoară în mod neinvaziv rigiditatea țesuturilor, steatoza și inflamația prin determinarea rigiditatii, atenuarii, vitezei sunetului și a viscozității. Prin aceasta tehnica se generează un impuls de împingere a forței de radiație acustică și se monitorizează propagarea undelor de forfecare rezultate în mai multe puncte. Viteza undei de forfecare (SWS) (în m/s) sau modulul Young calculat (kPA) este afișată în imagini 2D-SWE, oferind biomarkeri în timp real ai fibrozei hepatice. Scanarea SWE va fi efectuată folosind un sistem cu ultrasunete Aixplorer (Supersonic Mach 30) cu o sondă abdominală convexă (NAFLD Activity Score (NAS) –1).<br><br>8. **Biopsia hepatică percutanată (PLB) sau Biopsia hepatică transjugulară (TJLB)** va fi efectuată pentru diagnosticul NAFLD (ca si standard de aur”), la alegere, conform ghidurilor și caracteristicilor pacientului, sub ghidaj ecografic, sau videofluoroscopie cu raze X, respectiv la Cabinet Fibroscan și Laboratorul de Hemodinamica Hepatica UMF Cluj, Clinica Medicala III (IRGH).<br><br>9. **Examen histologic**. Va fi efectuată de un singur anatomopatolog autorizat, folosind Componenta de evaluare a fibrozei a sistemului de

	stadializare NASH CRN, Scorul de activitate al NAFLD (NAS), scorul pentru steatoză, inflamație și balonare, sistemul de scor Brunt pentru NASH/NAFLD.

Table 1. Reference investigations for ULYSES project

Blood	Imaging, ultrasound, elastography	Hemodynamics	Histology	Other
**liver function**: bilirubin, albumin, transaminases, cholestasis<br>**renal function**: urea, creatinine, Na, K<br>**full blood cell count**<br>**coagulation**: INR, PT<br>**inflammation**: CRP, PCT, calprotectin, TNFα, Il-6, Il-8, ICAM1, VCAM1, fibrinogen<br>**apoptosis/necrosis**: cytokeratin-18<br>**metabolic/liver function**: apolipoprotein A1<br>**metabolic parameters**: total cholesterol, triglycerides, HDL, LDL, glucose level, insulin level, HOMA-IR (insulin, glucose)<br>**hepatocyte markers**: ASGPR1, SLC27A5<br><br>*Na- sodium, K-potassium, INR-international normalized radio, PT-prothrombin time, CRP-C reactive protein, PCT-procalcitonin, TNFα-tumoral necrosis factor* α, Il-6 – Interleukin 6, Il-8 – Interleukin 8, HDL-high density cholesterol, LDL-low density cholesterol, HOMA-IR – homeostatic model assessment for insulin resistance, ICAM1- intercellular adhesion molecule 1, VCAM1-vascular adhesion molecule 1, ASGPR1 -aialoglycoprotein receptor 1, SLC27A5 -solute carrier family 27 member 5	**ultrasonography** (FLL), **VCTE** (LS, CAP)**, 2D-SWE** (LS, attenuation and speed of sound, liver viscosity).<br><br>**portal vein**: velocity, diameter, thrombosis<br>**HV**: diameter, flow, velocity<br>**HA**: RI<br><br>**Fast score** (CAP (dB/m), LS(kPa), AST(U/L)<br>**Agile 4+ score** *(AST(U/L, ALT(U/L), PLT(10*9/L), LS(kPa), diabetes, gender, age*<br><br>*FLL-focal liver lesions, LS – liver stiffness, CAP-controlled attenuation parameter, 2D-SWE- 2D share wave elastography, HV-hepatic vein, RI-resistivity index, AST-aspartate aminotransferase, ALT – alanin aminotransferase, PLT – platelets, VCTE – vibration controlled transient elastography*	**liver**: FHVP, WHVP, HVPG, IVCP<br><br>*FHVP- free hepatic vein pressure, WHVP-wedged hepatic vein pressure, HVPG-hepatic vein pressure gradient, IVCP-inferior vena cava pressure*	**liver**: NAS, steatosis, inflammation, ballooning, NASH CRN<br><br>*NAS – NAFLD activity score, NASH CRN – NASH clinical research network scoring system*	**anthropometric and demographic data**<br><br>**quality of life scores**<br><br>**arterial blood pressure** (3 measures)<br><br>**Child-Pugh and MELD** scores for cirrhotic NASH<br><br>**fibrosis scores**<br>**FIB-4** (age, transaminases, thrombocytes)<br>**NASH test** (age, body mass index, transaminases, albumin, glucose tolerance/diabetes)<br>**ELF** test- hyaluronic acid (HA), procollagen III amino-terminal peptide (PIIINP), and tissue inhibitor of matrix metalloproteinase 1 (TIMP-1)<br>FibroTest<br><br>*MELD- model for end stage liver disease, ELF – enhanced liver fibrosis,*

Table 2. Inclusion and exclusion criteria for ULYSES project

Inclusion criteria	Exclusion criteria
signed informed consent, patients that undertook liver biopsy, males and non-pregnant, non-lactating females between 18-80 years of age	chronic HBV infection (HBsAg positive), chronic HCV infection (HCV Ab and HCV RNA positive), subjects cured of HCV infection less than 5 years, other causes of liver disease including, but not limited to, alcoholic liver disease, hepatitis B, hepatitis C, autoimmune disorders (e.g., primary biliary cholangitis, primary sclerosing cholangitis, and autoimmune hepatitis), drug-induced hepatotoxicity, Wilson disease, iron overload, alpha-1-antitrispin deficiency, based on medical history and/or centralized review of liver histology, history of liver transplantation, current alcohol consumption greater than 30 g/day for males or 20 g/day for females

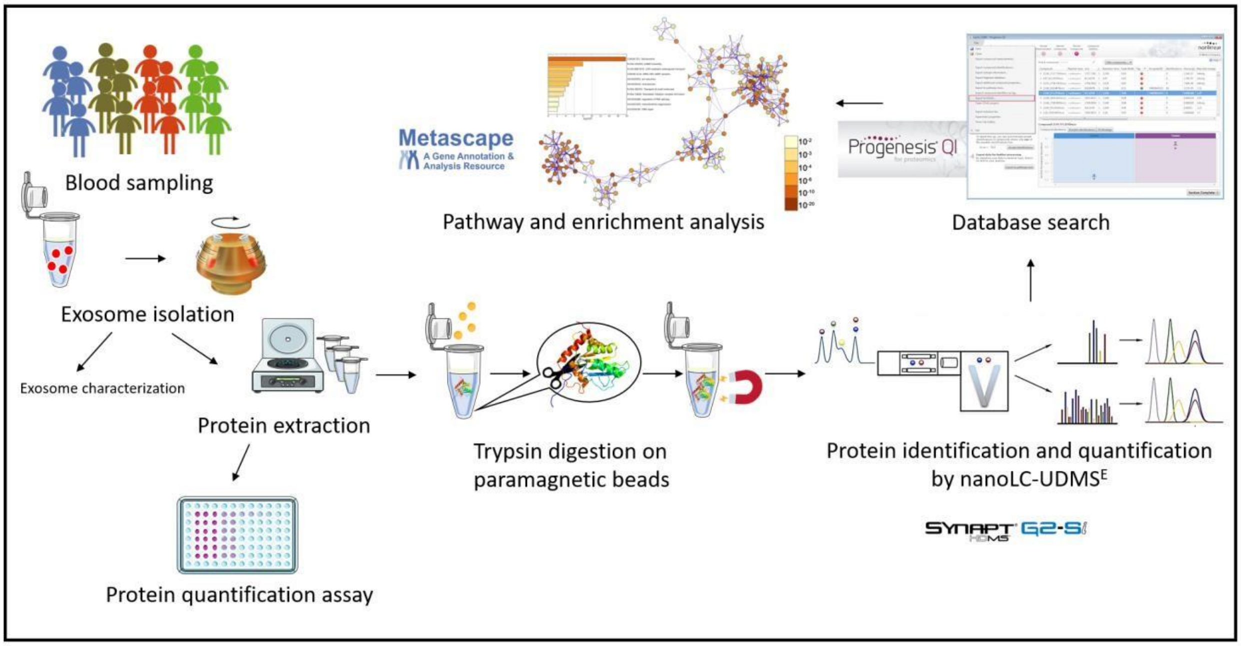

Figure 1. Providing the “proteomic” signature in NAFLD

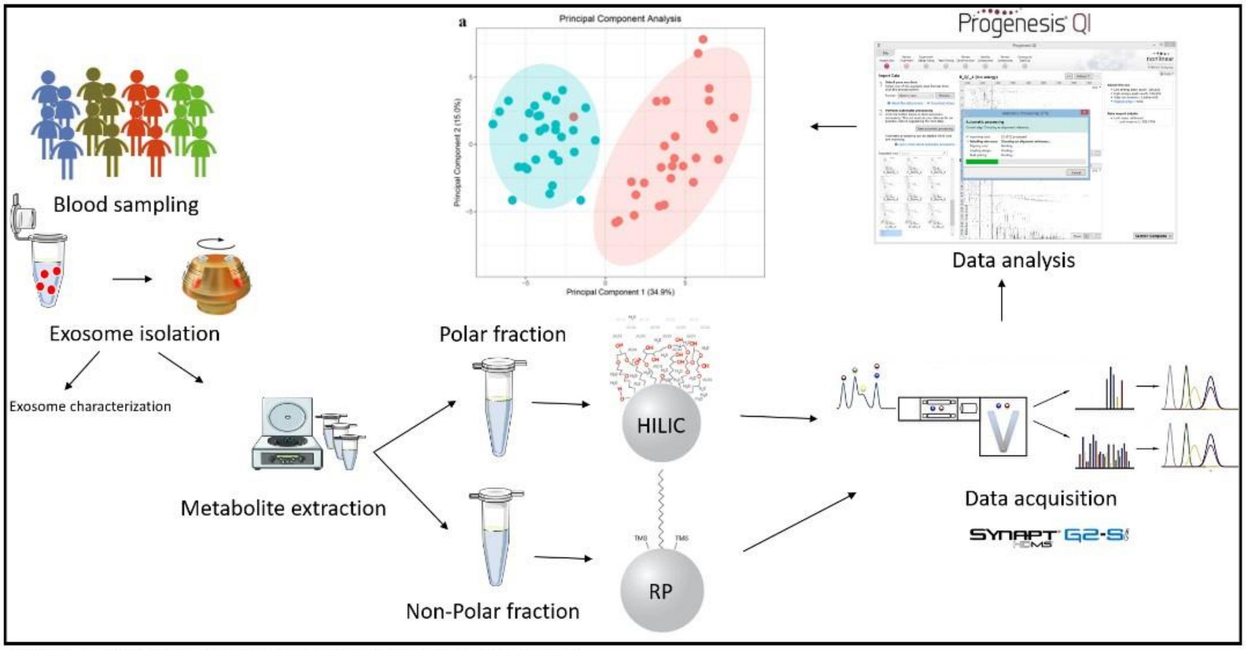

Figure 2. Providing the “metabolomic” signature in NAFLD

Referințe:

1. Estes C, Razavi H, Loomba R, Younossi Z, Sanyal AJ. Modeling the epidemic of nonalcoholic fatty liver disease demonstrates an exponential increase in burden of disease. Hepatology. 2018 Jan;67(1):123-33.

2. Ozturk A, Mohammadi R, Pierce TT, Kamarthi S, Dhyani M, Grajo JR, Corey KE, Chung RT, Bhan AK, Chhatwal J, Samir AE. Diagnostic Accuracy of Shear Wave Elastography as a Non-invasive Biomarker of High-Risk Non-alcoholic Steatohepatitis in Patients with Non-alcoholic Fatty Liver Disease. Ultrasound in medicine & biology. 2020 Apr 1;46(4):972-80.

3. Mittal S, El-Serag HB, Sada YH, Kanwal F, Duan Z, Temple S, May SB, Kramer JR, Richardson PA, Davila JA. Hepatocellular carcinoma in the absence of cirrhosis in United States veterans is associated with nonalcoholic fatty liver disease. Clinical Gastroenterology and Hepatology. 2016 Jan 1;14(1):124-31.

4. Dasarathy S, Dasarathy J, Khiyami A, Joseph R, Lopez R, McCullough AJ. Validity of real time ultrasound in the diagnosis of hepatic steatosis: a prospective study. Journal of hepatology. 2009 Dec1;51(6):1061-7.

5. Bercoff J, Tanter M, Fink M. Supersonic shear imaging: a new technique for soft tissue elasticity mapping. IEEE transactions on ultrasonics, ferroelectrics, and frequency control. 2004 Aug 9;51(4):396-409.

6. Petroff D, Blank V, Newsome PN, Voican CS, Thiele M, de Lédinghen V, Baumeler S, Chan WK, Perlemuter G, Cardoso AC, Aggarwal S. Assessment of hepatic steatosis by controlled attenuation parameter using the M and XL probes: an individual patient data meta-analysis. The Lancet Gastroenterology & Hepatology. 2021 Mar 1;6(3):185-98.

7. Abels ER, Breakefield XO. Introduction to extracellular vesicles: biogenesis, RNA cargo selection, content, release, and uptake. Cellular and molecular neurobiology. 2016 Apr 1;36(3):301-12.

8. Chen L, Chen R, Kemper S, Brigstock DR. Pathways of production and delivery of hepatocyte exosomes. Journal of cell communication and signaling. 2018 Mar;12(1):343-57.

9. Zhao Y, Zhao MF, Jiang S, Wu J, Liu J, Yuan XW, Shen D, Zhang JZ, Zhou N, He J, Fang L. Liver governs adipose remodelling via extracellular vesicles in response to lipid overload. Nature communications. 2020 Feb 5;11(1):1-7.

10. Jiang F, Chen Q, Wang W, Ling Y, Yan Y, Xia P. Hepatocyte-derived extracellular vesicles promote endothelial inflammation and atherogenesis via microRNA-1. Journal of hepatology. 2020 Jan1;72(1):156-66.

11. Povero D, Yamashita H, Ren W, Subramanian MG, Myers RP, Eguchi A, Simonetto DA, Goodman ZD, Harrison SA, Sanyal AJ, Bosch J. Characterization and proteome of circulating extracellular vesicles as potential biomarkers for NASH. Hepatology communications. 2020 Sep;4(9):1263-78.

12. Lourenço C, Constâncio V, Henrique R, Carvalho Â, Jerónimo C. Urinary Extracellular Vesicles asPotential Biomarkers for Urologic Cancers: An Overview of Current Methods and Advances. Cancers.2021 Jan;13(7):1529.

13. Constâncio V, Barros-Silva D, Jerónimo C, Henrique R. Known epigenetic biomarkers for prostate cancer detection and management: exploring the potential of blood-based liquid biopsies. Expert review of molecular diagnostics. 2019 May 4;19(5):367-75.

14. Ruivo CF, Adem B, Silva M, Melo SA. The biology of cancer exosomes: insights and new perspectives. Cancer research. 2017 Dec 1;77(23):6480-8.

15. Ando Y, Jou JH. Nonalcoholic fatty liver disease and recent guideline updates. Clinical Liver Disease. 2021 Jan;17(1):23.

16. Royo F, Gil-Carton D, Gonzalez E, Mleczko J, Palomo L, Perez-Cormenzana M, Mayo R, Alonso C, Falcon-Perez JM. Differences in the metabolite composition and mechanical properties of extracellular vesicles secreted by hepatic cellular models. Journal of extracellular vesicles. 2019 Dec 1;8(1):1575678.

17. Cuykx M, Negreira N, Beirnaert C, Van den Eede N, Rodrigues R, Vanhaecke T, Laukens K, Covaci A. Tailored liquid chromatography–mass spectrometry analysis improves the coverage of the intracellular metabolome of HepaRG cells. Journal of Chromatography A. 2017 Mar 3;1487:168-78.
