# Supplementary material for: FAST and Agile–the MASLD drift: Validation of Agile 3+, Agile 4 and FAST scores in 246 biopsy-proven NAFLD patients meeting MASLD criteria of prevalent caucasian origin
Source: PLoS One. 2024 May 23;19(5):e0303971. doi: 10.1371/journal.pone.0303971 (PMC11115280; doi:10.1371/journal.pone.0303971)
Supplement: Attachment — Submitted filename: Response to Reviewers cor MLP .docx [file pone.0303971.s003.docx]

Dear Reviewers,

Thank you sincerely for your constructive feedback. We greatly appreciate your insights. Below, you will find our response addressing your comments:

**Response for Reviewer 1**:

1. Line 380. From our cohort of biopsy-proven NAFLD patients, out of 256 patients with reliable VCTE measurements, 246 (98.4%) met the criteria for MASLD definition, ….The number of patients with reliable VCTE should be 250.

**Response**: Thank you for pinpointing this error We corrected the statement. The correct version: 252 (98.4%) met the criteria for MASLD.

**Response for Reviewer 2:**

1. The patients in this study were enrolled from 2007 to 2023, which is a long period. The accuracy of the measurement of Fibroscan was a concern since the machine had been remodeled afterward. Please describe this issue and its limitations in the discussion.

**Response**: Thank you for the comment. We added in the discussions the following paragraph: “Another limitation of the study could be the extended duration over which the analysis was conducted (2007-2023) and the fact that, during this period, we utilized 2 different Fibroscan devices (FibroScan X1115305 from the begining, respectively FibroScan® Expert 630 starting with 2016). As the number of biopsies for NAFLD patients from our tertiary center was not very large, we aimed to include as many probes as possible in our analysis, even if at some point, a new variant of the equipment was used. Nevertheless, the protocol has been rigorously followed since its introduction until the present.

2. Please compare the difference between the cut-off value proposed in this study and the recommended cut-off from the literature. After your analysis, what will be the cut-off value recommended for clinical suggestions?

**Response**: Thank you for the comment. We added in the “Discussions” section the following paragraph: “For Agile 3+, our thresholds - 0.480 for ruling out (Se ≥90%) and 0.680 for ruling in (Sp ≥90%) advanced fibrosis - closely mirrored the standard thresholds of 0.451 and 0.679, respectively. Similarly, for Agile 4, our thresholds - 0.380 for ruling out (Se ≥90%) and 0.520 for ruling in (Sp ≥90%) cirrhosis - were in line with the literature's proposed thresholds of 0.251 and 0.565, respectively. Given the excellent diagnostic performance demonstrated by the standard cut-offs in our cohort, they can be reliably applied within the Caucasian population”.

3. Because the Agile 3+, Agile 4, and Fast scores identify populations with different fibrotic and inflammation statuses, the authors should provide an algorithm for patients to use the Agile and Fast score as a screening workflow for fibrosis and fibrotic NASH.

**Response**: Thank you for the comment. We added in the “Discussions” section the following paragraph: “Given that Agile 3+, Agile 4, and FAST scores identify populations with varied fibrotic and inflammatory statuses, they hold promise for inclusion in algorithms as a screening tool for fibrosis and fibrotic NASH (MASH) (29). We believe that they could serve as pivotal components within a clinical pathway, perhaps in a “secondary step”, as part of evaluations conducted within specialized medical centers. However, it is essential to recognize that these scores include laboratory tests such as platelets, AST, and ALT, commonly encompassed in the scores that are usually applied in a “first step” like FIB-4 or NAFLD fibrosis score. In this respect, we believe that the performance of a multistep algorithm including Agile 3+, Agile 4, or FAST scores should be carefully evaluated and validated in future studies.”

4. Please propose a recommendation for patients in the grey zone. Do they need additional monitoring to determine their real fibrosis status?

**Response**: Thank you for the comment. We added in the “Discussions” section the following paragraph: “As part of the clinical evaluation, especially for risk stratification, the patients that are left in the “grey zone” should undergo, in our opinion, additional monitoring to determine their real fibrosis status. In this scenario, the causes for false positives should be considered and another non-invasive test could be applied (ELF^TM^, FibroMeter^TM^, FibroTest^®)^ or the patient could undergo liver biopsy in case of discordant NITs”.

5. Because liver inflammation (ALT elevation) may impact the LSM-VCTE results. The authors are encouraged to do a subgroup analysis for the performance of the FAST score stratified by ALT levels (eg. ALT < 1x ULN vs. ALT >= 1X ULN).

**Response**: Thank you for the comment. We included the subgroup analysis in the “Results” section. “In the subgroup analysis for FAST Score, the AUROCs were 0.70 [0.59, 0.80] for ALT ≥35 UI/L and 0.60 [0.42, 0.77] for ALT <35 UI/L, respectively”.
